# Sutton on Scrofula

**Published:** 1846-12

**Authors:** 


*Sutton on Scrofula.*—The Western Journal of Medicine and Surgery publishes the Essay of W. L. Sutton, M. D., of Georgetown, Ky., on Scrofula, to which was awarded recently the prize offered by the Medical Society of Tennessee. We subjoin the general conclusions of the writer.—*Ed. Buff. Jour.*

"*Conclusions.*—After such reflections as I have been able to make, I arrive at the following conclusions:

1st. That in a vast majority of instances, scrofula owes its existence to inheritance; yet

2d. That there is no absolute necessity that a child having a scrofulous parent shall be scrofulous; on the contrary,

3d. That when one parent is scrofulous and the other not, a child which resembles the scrofulous parent will be much more apt to have scrofula than the one which resembles the other parent; in fact, that the latter may have a well-grounded hope of escape.

4th. That the liability by inheritance depends upon a general, not upon a specific law, which is applicable to other diseases besides scrofula.

5th. That whilst a child born of scrofulous parents may escape, one born of parents not at all scrofulous, may have the disease.

6th. That scrofula depends upon an undue preponderance of the white parts of the blood, and the white tissues in the body.

7th. That in our treatment we should endeavor to restore a due proportion of the red particles of the blood, and of the red tissues to the body.

8th. That to effect this there is no specific, but we must be guided by general principles and rational views, precisely as is necessary to treat successfully any other disease.

9th. That how important soever medicine may be in the management of the disease, hygienic rules are by no means less so."

